# Styrene–Maleic Acid Copolymer-Based Nanoprobes for Enhanced Boron Neutron Capture Therapy

**DOI:** 10.3390/pharmaceutics17060738

**Published:** 2025-06-04

**Authors:** Mingjie Zhang, Shanghui Gao, Kai Yang, Benchun Jiang, Wei Xu, Waliul Islam, Shinnosuke Koike, Yusei Kinoshita, Hiroto Nakayama, Jianrong Zhou, Kazumi Yokomizo, Jun Fang

**Affiliations:** 1 Faculty of Pharmaceutical Sciences, Sojo University, Ikeda 4-22-1, Nishi-ku, Kumamoto 860-0082, Japan; zhangmingjie1101@126.com (M.Z.); gaoshanghui94@gmail.com (S.G.); yangkai@ahyz.edu.cn (K.Y.); jiangbenchun@126.com (B.J.); g1951043@m.sojo-u.ac.jp (S.K.); g1951039@m.sojo-u.ac.jp (Y.K.); trhs0515@icloud.com (H.N.); zhoujr@ph.sojo-u.ac.jp (J.Z.); yoko0514@ph.sojo-u.ac.jp (K.Y.); 2 Department of General Surgery, Shengjing Hospital of China Medical University, Shenyang 110004, China; 3 School of Pharmacy, Anhui Medical College, Hefei 230601, China; 4 Department of Medical Technology, Anhui Medical College, Hefei 230601, China; 5 Department of Gastrointestinal Surgery, Shengjing Hospital of China Medical University, Shenyang 110004, China; 6 Faculty of Advanced Science and Technology, Kumamoto University, Kumamoto 860-8555, Japan; xuwei@kumamoto-u.ac.jp; 7 Department of Microbiology, Graduate School of Medical Sciences, Kumamoto University, Kumamoto 860-8556, Japan; bcmb.waliul@gmail.com; 8 Department of Toxicology, School of Public Health, Anhui Medical University, Hefei 230022, China

**Keywords:** boron neutron capture therapy, nanoprobe, tumor targeting, EPR effect, styrene–maleic acid copolymer

## Abstract

**Background/Objectives**: Boron neutron capture therapy (BNCT) is a promising, less-invasive anticancer treatment. However, the development of effective boron-based agents (BNCT probes) remains a critical and challenging issue. Previously, we developed a styrene–maleic acid (SMA) copolymer conjugated with glucosamine, encapsulating boronic acid, which exhibited tumor-targeted distribution via the enhanced permeability and retention (EPR) effect. Building upon this approach, in this study, we designed and synthesized a series of SMA-based polymeric probes for BNCT and evaluated their biological activities, with a particular focus on tumor-targeting properties. **Methods**: Two SMA-based BNCT nanoprobes, SMA–glucosamine conjugated Borax (SG@B) and SMA-conjugated aminophenylboronic acid encapsulating tavaborole (S-APB@TB), were designed and synthesized. The boron content in the conjugates was quantified using inductively coupled plasma mass spectrometry (ICP-MS), while particle sizes were measured via dynamic light scattering (DLS). In vitro cytotoxicity was assessed using the MTT assay in mouse colon cancer C26 cells. The tissue distribution of the conjugates was analyzed in a mouse sarcoma S180 solid tumor model using ICP-MS. **Results**: Both SG@B and S-APB@TB formed nanoformulations with average particle sizes of 137 nm and 99 nm, respectively. The boron content of SG@B was 2%, whereas S-APB@TB exhibited a significantly higher boron content of 14.4%. Both conjugates demonstrated dose-dependent cytotoxicity against C26 cells, even in the absence of neutron irradiation. Notably, tissue distribution analysis following intravenous injection revealed higher boron concentrations in plasma and tumor tissues compared to most normal tissues, with S-APB@TB showing particularly favorable tumor accumulation. **Conclusions**: These findings highlight the tumor-targeting potential of SMA-based BNCT nanoprobes. Further investigations are warranted to advance their clinical development as BNCT agents.

## 1. Introduction

Boron Neutron Capture Therapy (BNCT) is a unique form of radiation therapy that offers highly selective destruction of cancer cells at the cellular level [1,2]. It is based on the nuclear reaction that occurs when non-radioactive boron-10 (^10^B), selectively accumulated in tumor cells, captures low-energy thermal neutrons, resulting in the emission of high linear energy transfer α-particles and lithium nuclei [1,2]. These particles have a very short path length (5–9 µm), equivalent to the diameter of a single cell, enabling localized cytotoxic effects while sparing surrounding healthy tissues. BNCT has shown promising results in the treatment of recurrent head and neck cancers, glioblastoma, and melanoma, particularly in cases where conventional therapies are limited [2]. As a biologically targeted radiotherapy, BNCT represents a powerful modality for precision oncology, combining molecular targeting with the physical advantages of particle therapy [1,2].

The therapeutic efficacy of BNCT relies on two critical factors: the preferential delivery and retention of boron compounds in tumor tissues, and an adequate thermal neutron flux at the tumor site. While the development and availability of specialized neutron sources are essential, the design of boron compounds with enhanced tumor-targeting capabilities is equally imperative for the success of BNCT. Currently approved boron delivery agents, such as boronophenylalanine (BPA) and sodium borocaptate (BSH), are small-molecule compounds [2]. Although they demonstrate moderate tumor affinity, they face significant limitations, of which the major concerns are the short plasma half-life and non-specific distribution to normal tissues. These drawbacks lead to suboptimal tumor accumulation and retention, while also increasing the risk of adverse effects due to off-target boron delivery. Therefore, there is an urgent need to develop next-generation boron compounds with significantly improved tumor selectivity and accumulation profiles.

In this context, nano-engineered boron compounds—such as polymer conjugates, polymeric micelles, nanoparticles, and liposomes—have recently garnered significant attention and are advancing rapidly in the field [3,4,5,6,7,8,9,10]. This strategy leverages a unique phenomenon observed in most solid tumors, known as the enhanced permeability and retention (EPR) effect [11,12,13]. Due to their abnormal vasculature and pathological characteristics, tumor tissues exhibit higher vascular permeability compared to normal tissues. Tumor blood vessels are typically immature, with wide gaps between endothelial cells, and are rich in vascular mediators such as bradykinin, nitric oxide, and vascular endothelial growth factor, all of which contribute to increased vascular permeability [10]. Furthermore, the lymphatic drainage system in tumor tissues is often impaired, leading to prolonged retention of macromolecules [12]. As a result, large molecules or nanocarrier systems—such as proteins, polymers, and liposomes—tend to preferentially accumulate in tumor tissues and remain there for extended periods, while showing limited distribution in normal tissues [11,12,13]. Nanomedicine based on the EPR effect is thus emerging as a promising approach for targeted cancer therapy, and it represents a significant recent advancement in the design of boron delivery systems for BNCT.

Based on the EPR effect, in our laboratory, we have developed polymeric nanomedicines for the treatment of cancer and inflammatory diseases [14]. More recently, we synthesized a polymer complex of boric acid using styrene-maleic acid copolymer (SMA) conjugated glucosamine (SGB), which showed tumor selective accumulation, the tumor concentration of boron after intravenous (i.v.) injection being much higher than that of normal tissues [15], indicating its potential as a candidate agent for BNCT. However, the boron content in this polymeric complex was about 1.5% (*w*/*w*) [15]. Given that a high amount of boron in tumor tissue is preferential in a clinical setting, in this study, we challenged ourselves to develop a series of polymeric boron compounds by utilizing SMA and different types of boron-containing molecules. This study was mostly focused on the synthesis and physicochemical characterization of polymeric boron compounds, especially focusing on their in vivo biodistribution, and the in vitro cytotoxicity of these compounds was also investigated.

## 2. Materials and Methods

### 2.1. Chemicals

SMA (average molecular weight: 1600) was obtained from Sigma (St. Louis, MO, USA). D-(+)-Glucosamine, sodium tetraborate decahydrate (borax), *p*-dimethylaminobenzaldehyde, 3-aminophenylboronic acid (APB), and sulfuric acid were purchased from Wako Pure Chemical Industries (Osaka, Japan). Tavaborole (TB) was obtained from TCI (Tokyo, Japan). 3-(4,5-Dimethylthiazol-2-yl)-2,5-diphenyltetrazolium bromide (MTT) was purchased from Dojindo Laboratories (Kumamoto, Japan). All other reagents and solvents were of reagent grade and used without further purification.

### 2.2. Synthesis of SMA–Glucosamine Conjugate (SG), and Its Complex with Borax (SG@B)

SMA (500 mg, 2.5 mmol) was dissolved in *N*,*N*-dimethylformamide (DMF), followed by the addition of glucosamine (837.5 mg, 5 mmol) and ethylamine (2.1 mL, 15 mmol). The reaction mixture was stirred overnight at 60 °C. After completion, the reaction mixture was dialyzed using a dialysis membrane with a molecular weight cut-off of 8000 Da (Wako) against deionized water (three cycles). The resulting SMA–glucosamine conjugate (SG) was collected by freeze-drying.

The glucosamine content in SG was quantified using the Elson–Morgan method [16]. Briefly, standard glucosamine solutions (0.1–1 mg/mL) and SG samples were prepared. To 0.5 mL of each sample, 0.1 mL of sodium carbonate solution (0.5 M) was added, followed by acetylacetone treatment. The mixture was heated in a water bath at 50 °C for 5 min. Ehrlich’s reagent (prepared by dissolving 500 mg of *p*-dimethylaminobenzaldehyde in 50 mL of ethanol and 50 mL of concentrated HCl) was then added, and the absorbance at 550 nm was measured after 5 min. The glucosamine content in SG was calculated using a standard calibration curve.

To prepare the SG@B complex, SG (60 mg) was dissolved in 12 mL of sodium carbonate solution (0.1 M), and borax (99 mg) was added. The mixture was stirred at room temperature for 24 h. After dialysis against deionized water and freeze-drying, the SG@B complex was obtained as a powder. The boron content in SG@B was quantified by inductively coupled plasma mass spectrometry (ICP-MS) in 1% HNO_3_, following the manufacturer’s instructions (Agilent7900, Agilent, Santa Clara, CA, USA). The results were calculated by comparison with a boron standard solution containing both ^10^B and ^11^B, and the total boron content is expressed as weight percent, representing the weight of boron relative to the total weight of the compound.

The particle size and zeta potential of the SG@B complex were determined by dynamic light scattering (DLS) and electrophoretic mobility measurements, respectively. For DLS analysis, SG@B samples were dissolved in 0.1 M bicarbonate buffer (pH 8.8) at a concentration of 15 mg/mL and filtered through a 0.2 μm membrane prior to measurement at 25 °C. Particle size was measured using a Model ELSZ-2000ZS electrophoretic light-scattering analyzer (Otsuka Electronics Co., Ltd., Osaka, Japan) equipped with a He/Ne laser, applying both cumulant and histogram analysis methods. Zeta potential was measured at 20 mg/mL in deionized water to evaluate surface charge changes before and after glucosamine conjugation to SMA and subsequent complex formation.

### 2.3. Synthesis of SMA-APB Conjugate (S-APB), and Its Micelle Formation with TB

SMA (300 mg, 1.5 mmol) was dissolved in 10 mL of DMF, followed by the addition of 3-aminophenylboronic acid (APB; 410.82 mg, 3 mmol). Ethylamine (1.26 mL) was also added, and the mixture was stirred overnight at 60 °C. After the reaction, the product was dialyzed using a dialysis membrane with a molecular weight cut-off of 8000 Da (Wako) against deionized water (three cycles). The resulting SMA–APB conjugate (S-APB) was obtained as a powder by freeze-drying.

To confirm the successful conjugation, infrared (IR) spectroscopy was performed on dried S-APB powder using potassium bromide pellets and analyzed with an FT/IR-4200 spectrometer (Jasco Corp., Tokyo, Japan). The particle size of S-APB in aqueous solution was measured by dynamic light scattering (DLS), as described in Section 2.2.

For the preparation of S-APB micelle encapsulating TB (S-APB@TB), S-APB (100 mg) was dissolved in 10 mL of deionized water, and the pH was adjusted to 11. Tavaborole (TB; 20 mg), previously dissolved in 1 mL of DMSO, was added dropwise to the S-APB solution. The pH was then adjusted to 7.4, and the mixture was stirred at room temperature for 24 h. The resulting micellar dispersion was dialyzed against deionized water and freeze-dried to yield the S-APB@TB powder.

The boron content in both S-APB and S-APB@TB was quantified by ICP-MS, following the manufacturer’s protocol.

In a separate experiment, the changes in particle size of SG@B, S-APB, and S-APB@TB in aqueous solution after incubation at room temperature were measured by DLS.

### 2.4. Transmission Electron Microscopy (TEM) Analysis of SG@B, S-APB, and S-APB@TB

SG@B, S-APB, and S-APB@TB were each dissolved in PBS at a concentration of 5 mg/mL and transferred onto TEM grids. The grids were vacuum-dried overnight at room temperature and then analyzed using transmission electron microscopy (TEM, ELS-C10, Okenshoji Co., Ltd., Tokyo, Japan) at an accelerating voltage of 80 kV (JEM-1400 Plus; JEOL, Tokyo, Japan).

### 2.5. In Vitro Cytotoxicity Assay

In vitro cytotoxicity of the SMA nano-boron compounds and their precursors (i.e., SMA, SG, SG@B, S-APB, and S-APB@TB) was determined by using the MTT method with colon carcinoma C26 (a kind gift from Professor Yu Ishima, Kyoto Pharmaceutical University) and Vero cells (monkey kidney epithelial cells, ATCC CCL-81). Cells were cultivated in RPMI-1640 with 10 % fetal bovine serum (PBS, Nichirei Biosciences INC., Tokyo, Japan) at 37 °C under 5 % CO_2_. The cells (1 × 10^4^ cells/well) were plated in 96-well culture plates (each group consisted of 8 wells) and cultured overnight, and the test samples of indicated concentrations were then added to the cells. After 24 h, the MTT assay was then performed, and the toxicity was quantified by the percentage of surviving cells compared with that without drug treatment (control).

### 2.6. Intracellular Uptake of SG@B and S-APB@TB Compared with Free Borax

Mouse colon cancer C26 cells were used for the intracellular uptake study. C26 cells (2 × 10⁴ cells/well) were seeded into 24-well plates (each group consisted of 4 wells) and cultured overnight. Then, SG@B, S-APB@TB, or free borax was added to the cells at a boron concentration of 20 µg/mL, and the cells were incubated at 37 °C. Control cells were treated with PBS(-). At the indicated time points (4 h, 24 h, and 48 h), the culture medium was removed, and the cells were washed four times with PBS. Cells were then lysed using 0.1% Triton X-100, and the resulting suspensions were centrifuged at 3000× *g*. The supernatants were collected and diluted with 1% HNO_3_, followed by quantification of intracellular boron content using ICP-MS.

### 2.7. Tissue Distribution and Pharmacokinetics of the SG@B and S-APB@TB Following Intravenous (i.v.) Injection

Male ddY mice (6 weeks old) were purchased from SLC (Shizuoka, Japan) and housed under controlled conditions (22 ± 1 °C, 55 ± 5% relative humidity, 12 h light/dark cycle). All animal experiments were approved by the Animal Ethics Committee (approval no. 2023-P-005) and conducted in accordance with the Laboratory Protocol for Animal Handling at Sojo University (Kumamoto, Japan). The endpoint of the experiment was governed by the tumor volume (up to ~2000 mm^3^). The mice were sacrificed by euthanasia using isoflurane.

Mouse sarcoma S180 cells (2 × 10^6^), maintained by weekly intraperitoneal passage in ddY mice, were implanted subcutaneously into the dorsal skin of recipient mice to generate solid tumors. When tumor diameters reached 10–12 mm, the mice were randomly grouped; each group consisted of 4 mice, which, based on our experience, was sufficient to obtain statistically significant data while minimizing animal use. Mice with tumor sizes smaller than 10 mm or larger than 12 mm were excluded from this study. To minimize potential confounders, all mouse cages were placed in the same position on the shelf, and the mice were numbered according to the order of injection.

SG@B and S-APB@TB were administered via the tail vein at a dose of 15 mg/kg (boron equivalent), formulated in 8.4% sodium bicarbonate solution. For comparison, a separate group received free borax (15 mg/kg, boron equivalent). After 24 h, mice were euthanized, and blood samples were collected from the inferior vena cava. Serum was obtained by centrifugation. To remove residual blood from tissues, mice were perfused with 20 mL of saline.

One hundred mg each of tumor tissue and major organs—including liver, spleen, kidney, colon, lung, and muscle—was excised and placed in test tubes. A mixture of concentrated nitric acid and sulfuric acid (1:1, 0.25 mL) was added, and the samples were digested at 80 °C for 2 h. After cooling, 10 mL of deionized water was added to each tube, followed by vortexing. An aliquot (~1 mL) of each digested sample was analyzed for boron content using ICP-MS, and results were calculated by comparing with a boron standard solution and expressed in parts per billion (ppb).

For pharmacokinetic analysis, healthy ddY mice were intravenously administered SG@B, S-APB@TB, or free borax at the same boron dose (15 mg/kg). Blood samples were collected at 0, 2, 4, 8, and 24 h post-injection. Plasma was isolated by centrifugation and subjected to acid digestion, followed by boron quantification using ICP-MS as described above.

### 2.8. Statistical Analysis

All data are presented as means ± standard error of the mean (SEM). Statistical analyses were performed using SPSS version 23.0 (IBM Corp., Armonk, NY, USA). Differences between the two groups were evaluated using Student’s *t*-test. For comparisons among multiple groups, one-way analysis of variance (ANOVA) was performed, followed by the Student–Newman–Keuls post hoc test. A *p*-value of less than 0.05 was considered statistically significant.

## 3. Results

### 3.1. Synthesis and Physicochemical Characterization of SMA–Glucosamine–Borax Complex

Building upon our previous study involving an SMA–glucosamine complex with boric acid [13], we synthesized a similar polymer complex using borax as the boron source. The conjugation protocol was also modified by conducting the reaction in *N*,*N*-dimethylformamide (DMF), which improved the overall efficiency. Using this optimized procedure, the SMA–glucosamine (SG) conjugate was obtained with a yield of 52%, and the glucosamine content in the polymer was determined to be 31%, as quantified by the Elson–Morgan method.

**Figure 1:**
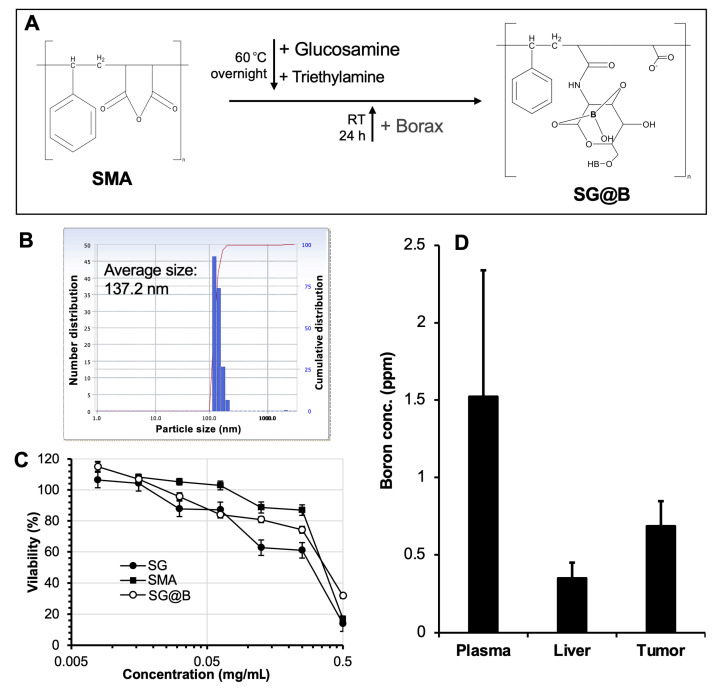
Synthesis and characterization of SMA–glucosamine–borax complex (SG@B). In (**A**), the brief reaction protocol is illustrated; it should be noted that the chemical structure of the final product, SG@B, is hypothetical. (**B**) shows the particle size of SG@B as measured by dynamic light scattering (DLS). In vitro cytotoxicity (**C**) and in vivo tissue distribution (**D**) were carried out using mouse colon cancer C26 cells. Data are expressed as Mean ± SE, n = 4–8. See text for details.

When borax was added to the solution of SMA–glucosamine, a boron-containing complex (SG@B) was formed (Figure 1A). DLS analysis confirmed the formation of nanoparticles with an average diameter of 137.2 nm (Figure 1B). However, TEM revealed irregularly shaped structures (Figure S1), suggesting that SG@B exists as a loosely associated complex or aggregate. This interpretation was partially supported by the observed changes in particle size over time: in aqueous solution, the DLS-measured size of SG@B increased progressively, with a marked enlargement noted after one week of incubation (Figure S2). The yield of SG@B was 78.3%, and the boron content, determined by ICP-MS, was 2%. Zeta potential analysis showed a surface charge of −38.19 mV, which was less negative than that of free SMA (Table 1). This reduction in surface charge indicates that glucosamine conjugation and subsequent complexation with borax partially neutralized the negative charge of SMA, consistent with our previous findings on the SGB complex [15].

### 3.2. Biological Evaluation of SG@B: In Vitro Cytotoxicity and In Vivo Body Distribution

For the biological evaluation, we assessed the cytotoxicity and biodistribution of the SG@B complex. In mouse colon cancer C26 cells, the cytotoxicity of SG@B (without irradiation) was compared to that of SG and free SMA. All samples exhibited dose-dependent cytotoxicity, with 50% inhibitory concentration (IC_50_) values ranging from 0.25 to 0.5 mg/mL (Figure 1C). Notably, SG and SG@B demonstrated stronger cytotoxic effects than free SMA, suggesting that glucosamine conjugation enhances tumoricidal activity. This finding is consistent with our previous report on the SGB complex [15]. Interestingly, in the normal Vero cell line, SG@B exhibited considerably lower cytotoxicity, with cell viability remaining at approximately 60% at a concentration of 0.5 mg/mL.

In a mouse sarcoma S180 solid tumor model, the boron content in the tumor, plasma, and liver was measured 24 h after intravenous (i.v.) injection of SG@B. The results, shown in Figure 1D, revealed higher boron accumulation in the tumor and plasma compared to the liver, indicating prolonged plasma circulation and tumor-targeting properties of the SG@B complex.

### 3.3. Design and Synthesis of SMA Complex Containing Tavaborole

**Figure 2:**
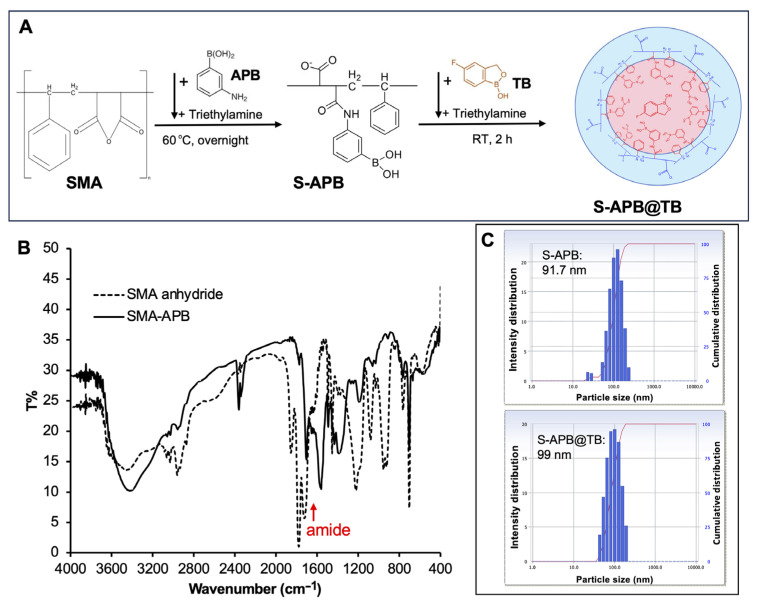
Synthesis and characterization of the SMA-APB conjugate (S-APB) and its micelle containing tavaborole (S-APB@TB): (**A**) illustrates the brief reaction protocol, with the chemical structures of the products presented as hypothetical images; (**B**) shows the IR spectra confirming the formation of the amide bond in SMA-APB; (**C**) presents the particle size of S-APB and S-APB@TB as measured by dynamic light scattering (DLS). See the text for further details.

To increase the boron content of the SMA–boron complex, we designed a micellar formulation of SMA–boron. As shown in Figure 2A, SMA was first conjugated with APB via an amide bond. The conjugation was confirmed by IR spectroscopy, where a new peak corresponding to the amide bond appeared after conjugation (Figure 2B). The SMA–APB conjugate (S-APB) exhibits amphiphilic properties, allowing it to self-assemble into micelles in aqueous solution, with an average particle size of 91.7 nm (Figure 2C). Zeta potential analysis revealed a surface charge of −36.23 mV, which was less negative than that of free SMA and similar to that of SG@B (Table 1).

To the aqueous solution of S-APB, tavaborole (TB), a clinically used antifungal drug containing boron, was added. The hydrophobic TB was incorporated into the core of the S-APB micelle, forming a stable micelle (S-APB@TB) with an average size of 99 nm (Figure 2C). The surface charge of S-APB@TB is −35.11 mV, which is slightly less negative than S-APB (Table 1), suggesting no significant influence on the surface charge of the micelle after encapsulation of TB.

TEM analysis revealed spherical structures for both S-APB and S-APB@TB (Figure S1), indicating micelle formation. Upon incubation at room temperature, the particle sizes in aqueous solution remained largely stable, with only slight increases observed after one week (Figure S2). This indicates significantly improved stability compared to SG@B, likely due to the formation of well-defined micellar structures.

### 3.4. In Vitro Cytotoxicity and Intracellular Uptake of S-APB@TB

**Figure 3:**
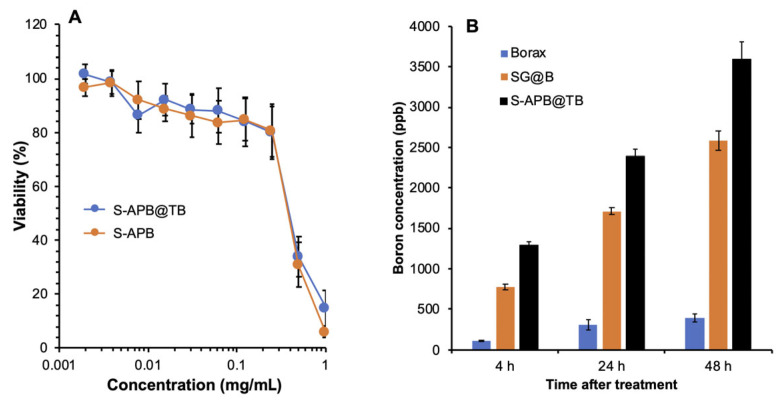
In vitro cold cytotoxicity (without neutron irradiation) and intracellular uptake of SG@B, S-APB, and S-APB@TB using colon cancer C26 cells. The cytotoxicity was investigated by using the MTT assay (**A**), and the intracellular uptake (**B**) was determined by quantifying the boron concentration in the cells treated by the indicated samples using ICP-MS. Data are expressed as Mean ± SE, n = 4–8. See text for details.

We next evaluated the in vitro cytotoxicity of S-APB@TB in C26 colon cancer cells under non-irradiated (cold) conditions. No significant cytotoxicity was observed for either S-APB@TB or S-APB at concentrations up to 0.25 mg/mL (Figure 3A). In contrast, SG and SG@B showed IC_50_ values around 0.25 mg/mL (Figure 1C), indicating that S-APB and S-APB@TB exhibit lower cytotoxicity. These findings further support the notion that glucosamine contributes significantly to the tumoricidal activity of the SG@B complex. Additionally, in the normal Vero cell line, neither S-APB@TB nor S-APB showed notable cytotoxicity at concentrations up to 0.5 mg/mL (Figure S3), demonstrating the favorable safety profile of these compounds.

We also compared the intracellular uptake of S-APB@TB with SG@B and borax. Both SG@B and S-APB@TB exhibited significantly higher boron uptake in C26 cells compared to borax (Figure 3B). Notably, the uptake of S-APB@TB was significantly higher than that of SG@B (Figure 3B), indicating the greater therapeutic potential of S-APB@TB.

### 3.5. In Vivo Body Distribution and Pharmacokinetics of S-APB@TB

**Figure 4:**
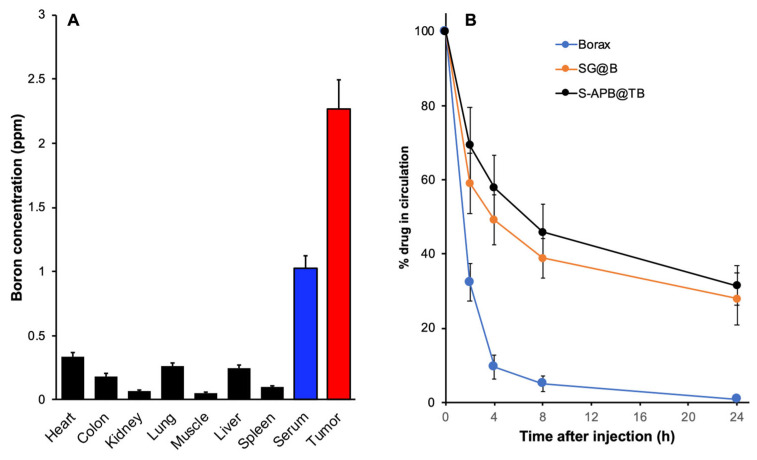
In vivo tissue distribution and pharmacokinetics of SG@B, S-APB, and S-APB@TB using mouse sarcoma S180 solid tumor model. In S180 tumor model, each sample was injected i.v. when the tumor reached 10–12 mm in diameter. For tissue distribution study (**A**), at 24 h after i.v. injection, mice were sacrificed, and each tissue, including the tumor, was collected, and the boron concentration in each tissue was quantified by ICP-MS. For pharmacokinetic study (**B**), at the indicated time after i.v. injection, mice were sacrificed and blood was collected; the boron concentration in the plasma was then quantified by ICP-MS. Data are expressed as Mean ± SE, n = 4. See text for details.

Finally, we investigated the in vivo pharmacokinetics of S-APB@TB. As shown in Figure 4A, in the S180 solid tumor model, at 24 h after i.v. injection, we observed that the boron content in the tumor was significantly higher compared to most normal tissues and serum, suggesting tumor-selective accumulation due to the EPR effect. Additionally, the circulation time of S-APB@TB in the bloodstream was much longer than that of the low molecular weight boron compound, Borax (Figure 4B). While SG@B also showed a prolonged circulation time compared to Borax, its plasma half-life was slightly shorter than that of S-APB@TB (Figure 4B).

## 4. Discussion

In this study, we aimed to develop nano-boron compounds with tumor-targeting potential for BNCT. We designed a series of polymeric boron complexes using the SMA copolymer. The results demonstrated that after adding glucosamine to SMA, boron could be efficiently incorporated to form a stable boron complex, SG@B, which showed tumor-targeted accumulation based on the EPR effect (Figure 1). Conjugation of SMA directly with a boron-containing molecule, APB, resulted in a nanoformulation, S-APB, with an average particle size of 91.7 nm, indicating the formation of a micelle structure (Figure 2). Further addition of TB into the S-APB micelle increased the particle size to 99 nm, suggesting the successful encapsulation of TB into the micelle. Consequently, a micellar formulation of the SMA–boron compound, S-APB@TB, was achieved with a significantly increased boron content (14.4%) (Figure 2, Table 1) and demonstrated relatively high tumor accumulation and prolonged plasma half-life (Figure 4).

Regarding the hydrodynamic size of SG@B, S-APB, and S-APB@TB, which are around 100 nm, we found that they are larger than the initial SMA micelle (10 nm) (Table 1). The increased size of SG@B may probably be due to the agglomeration or network-like association by the crosslinking via borate–diol complexation. Specifically, borax forms reversible covalent bonds with cis-diols on glucosamine, which may lead to intermolecular borate-diol complexation, where one borate anion can bridge two separate SMA–glucosamine chains or micelles to form larger aggregates or networks. In addition, the decreased surface negative charge (Table 1) might reduce electrostatic repulsion between micelles, thus promoting closer packing and agglomeration. For S-APB, we consider that the increased hydrophobicity after conjugation with APB likely contributes to this size increase. The shift in the amphiphilic balance favors micelle formation, leading to a larger micelle size. Furthermore, the phenyl rings in APB may undergo π–π stacking, promoting micelle association and the formation of larger assemblies. The addition of bulky side chains, such as aminophenylboronic acid, could also increase the coronal thickness of the micelles, contributing to the observed size increase. For S-APB@TB, the incorporation of the hydrophobic compound TB is presumed to result in its encapsulation within the micellar core, further contributing to the overall size. These hypotheses warrant further investigation to fully elucidate the mechanisms underlying the observed size increases.

Increasing the boron concentration in the tumor is critical for the success of BNCT. Effective BNCT generally requires ≥20 ppm of ^10^B in tumor tissue, while the surrounding normal tissues should contain <10 ppm, with a desired tumor-to-normal tissue ratio ≥3:1 [9]. These levels are necessary to ensure sufficient thermal neutron capture in the tumor while minimizing damage to normal tissues. Current clinical BNCT uses BPA or BSH as boron sources. The therapeutic regimen for BPA ranges from 300 to 900 mg/kg, achieving tumor boron concentrations of 15–30 ppm [17]. For BSH, the typical dosing is 100 mg/kg, which can result in tumor boron concentrations around 30 ppm [17]. While BPA needs a higher dose to reach the desired tumor blood concentration, it shows a higher tumor-selective uptake with tumor-to-normal tissue ratios exceeding 2.5:1; the BSH can result in higher tumor boron concentrations with a relatively lower dose, but with tumor-to-normal tissue ratios generally less than 1. However, in a study of 25 patients with glioblastoma multiforme or anaplastic astrocytoma, BSH was infused at doses of 15, 25, and 50 mg boron/kg body weight. For the 50 mg/kg dose, the mean tumor boron concentration was 11.9 μg/g, with tumor/normal brain tissue ratios of 3.8 for glioblastoma and 3.2 for anaplastic astrocytoma [18]. 

In the present study, we found that S-APB@TB, at a dose of 15 mg/kg, resulted in a tumor boron concentration of approximately 2.5 ppm 24 h after intravenous injection (Figure 4A). While this concentration is lower than the desired ≥20 ppm, it is reasonable and possible to achieve this target with a higher dose of S-APB@TB. Moreover, we observed tumor-to-normal tissue ratios greater than 5, at 6:1 (Figure 4A), suggesting high tumor selectivity and the potential for enhanced safety during treatment. The prolonged plasma half-life (Figure 4B) ensures sustained retention of the drug in the tumor tissue, further enhancing the BNCT effect.

After accumulation in the tumor tissue, the uptake of boron compounds by tumor cells is an essential step for effective BNCT. We compared the intracellular uptake of the SMA-based nanosystem of boron in cultured C26 colon cancer cells with that of the free boron compound, borax. As expected, SG@B and S-APB@TB exhibited significantly higher intracellular boron uptake compared to free borax (Figure 3B). This enhanced uptake is likely attributed to the hydrophobic nature of SMA, the encapsulation of TB, and the micelle formation, which allows rapid entry into cells through free diffusion (hydrophilic TB) and endocytosis (micelle). In contrast, the hydrophilic boron compound borax relies mostly on transporter-dependent internalization, resulting in relatively low uptake efficiency. In our previous study, we compared different polymeric nanosystems, including polyethylene glycol (PEG) and SMA, and found that SMA rendered higher intracellular uptake compared to the highly hydrophilic PEG, which was comparable to that of hydrophobic small molecules [19,20]. Moreover, SMA exhibits a high affinity for serum albumin, which binds to albumin in circulation and cell culture systems [11,21]. Previous studies have suggested that albumin-bound nanodrugs exhibit enhanced intracellular uptake [22,23,24]. Together, these findings, along with the prolonged plasma half-life (Figure 4B) and EPR-based tumor-targeted accumulation (Figure 4A), highlight the high therapeutic potential of the SMA-based nano-boron compound, warranting further investigation.

Among the polymeric nano-boron compounds, we observed stronger in vitro cytotoxicity for SG@B compared to others (Figure 1C). This effect may be attributed to the involvement of glucosamine in the system. Numerous studies have demonstrated the anticancer effect of glucosamine in both in vitro and in vivo models, with mechanisms including induction of cell death pathways, triggering oxidative stress and apoptosis, suppression of cell proliferation, and inhibition of angiogenesis [25,26]. In our previous study, we also observed the significant antitumor effect of the SMA–glucosamine boric acid complex, as well as its precursor, SMA–glucosamine conjugate, even without neutron irradiation [15]. The cytotoxicity of the SMA–glucosamine conjugate was found to be comparable to, or even greater than, that of free glucosamine [15]. Given that glucosamine is covalently linked to SMA via a relatively stable amide bond, which is not readily cleaved, it is presumed that little to no free glucosamine is released from the conjugate. Therefore, we hypothesize that the glucosamine moieties within the SMA–glucosamine conjugate retain functional activity similar to free glucosamine, while the SMA backbone enhances cellular uptake. This hypothesis warrants further investigation. Notably, the SMA–boron compounds exhibited minimal cytotoxicity in normal Vero cells (Figure S3), suggesting a favorable safety profile. However, the cold cytotoxicity (i.e., without neutron irradiation) observed with SG underscores the importance of thoroughly evaluating the toxicity profiles of these boron-containing compounds. This will be a key focus in future stages of development. Taken together, we anticipate that nano-boron compounds containing glucosamine could have a synergistic anticancer effect through BNCT and glucosamine-based mechanisms. Our follow-up study will focus on the utilization of the SG platform with high-boron-content compounds, such as o-carborane, to further enhance the boron content in tumor tissues and improve the therapeutic potential of BNCT.

In conclusion, polymeric nano-delivery systems of boron compounds based on SMA exhibit promising therapeutic potential for BNCT by leveraging the EPR effect-based tumor-selective accumulation, prolonged plasma half-life, and enhanced intracellular uptake. The therapeutic effect could be further augmented by incorporating glucosamine into the system. By modulating the nanoformulation and selecting high-content boron-containing compounds, we anticipate the development of next-generation BNCT compounds using the SMA-based nano-drug delivery system.

## Figures and Tables

**Table 1:** Physiochemical properties of SMA, SG@B, and S-APB@TB.

Properties	SMA	SG@B	S-APB	S-APB@TB
Hydrodynamic diameter by DLS	10 nm	137.2 nm	91.7 nm	99 nm
Arbitrary surface charge (zeta potential)	−46.24 mV	−38.19 mV	−36.23 mV	−36.11 mV
Boron content	―	2 % *	―	14.4 % *

* The boron content is expressed as wt/wt%.

## Data Availability

The original contributions presented in this study are included in the article. Further inquiries can be directed to the corresponding author.

## Supplementary Materials

The following supporting information can be downloaded at: https://www.mdpi.com/article/10.3390/pharmaceutics17060738/s1, Figure S1: Transmission electron microscopy (TEM) analysis of SG@B, S-APB, and S-APB@TB. Figure S2: Changes in particles of SMA boron compounds after incubation at room temperature. The particle sizes were measured by dynamic light scattering (DLS). Figure S3: In vitro cytotoxicity of SMA boron compounds in Vero cells.
