# Supplementary material for: Styrene–Maleic Acid Copolymer-Based Nanoprobes for Enhanced Boron Neutron Capture Therapy
Source: Pharmaceutics. 2025 Jun 4;17(6):738. doi: 10.3390/pharmaceutics17060738 (PMC12196042; doi:10.3390/pharmaceutics17060738)
Supplement: Supplementary file 1 [file pharmaceutics-17-00738-s001.zip › pharmaceutics-3648647-supplementary.pdf]

Article

Styrene–Maleic Acid Copolymer-Based Nanoprobes for Enhanced Boron Neutron Capture Therapy

Mingjie Zhang [1,2,†], Shanghui Gao [1,3,†], Kai Yang [1,4], Benchun Jiang [1,5], Wei Xu [6], Waliul Islam [7,‡], Shinnosuke Koike [1], Yusei Kinoshita [1], Hiroto Nakayama [1], Jianrong Zhou [1], Kazumi Yokomizo [1] and Jun Fang [1,8,*]

[1] Faculty of Pharmaceutical Sciences, Sojo University, Ikeda 4-22-1, Nishi-ku, Kumamoto 860-0082, Japan; zhangmingjie1101@126.com (M.Z.); gaoshanghui94@gmail.com (S.G.); yangkai@ahyz.edu.cn (K.Y.); jiangbenchun@126.com (B.J.); g1951043@m.sojo-u.ac.jp (S.K.); g1951039@m.sojo-u.ac.jp (Y.K.); trhs0515@icloud.com (H.N.), zhoujr@ph.sojo-u.ac.jp (J.Z.), yoko0514@ph.sojo-u.ac.jp (K.Y.)
[2] Department of General Surgery, Shengjing Hospital of China Medical University, Shenyang 110004, China
[3] School of Pharmacy, Anhui Medical College, Hefei 230601, China
[4] Department of Medical Technology, Anhui Medical College, Hefei 230601, China
[5] Department of Gastrointestinal Surgery, Shengjing Hospital of China Medical University, Shenyang 110004, China
[6] Faculty of Advanced Science and Technology, Kumamoto University, Kumamoto 860-8555, Japan; xuwei@kumamoto-u.ac.jp
[7] Department of Microbiology, Graduate School of Medical Sciences, Kumamoto University, Kumamoto 860-8556, Japan; bcmb.waliul@gmail.com
[8] Department of Toxicology, School of Public Health, Anhui Medical University, Hefei 230022, China
* Correspondence: fangjun@ph.sojo-u.ac.jp; Tel.: +81-96-326-4137
† These authors equally contribute to this paper.
‡ Current address: Division of Gastroenterology and Hepatology, Johns Hopkins University School of Medicine, 720 Rutland Ave., Baltimore, MD 21205, USA.

Supplementary Materials

Contents:

Figure S1. Transmission electron microscopy (TEM) analysis of SG@B, S-APB, and S-APB@TB.

Figure S2. Changes in particles of SMA boron compounds after incubation at room temperature. The particle sizes were measured by dynamic light scattering (DLS).

Figure S3. In vitro cytotoxicity of SMA boron compounds in Vero cells.

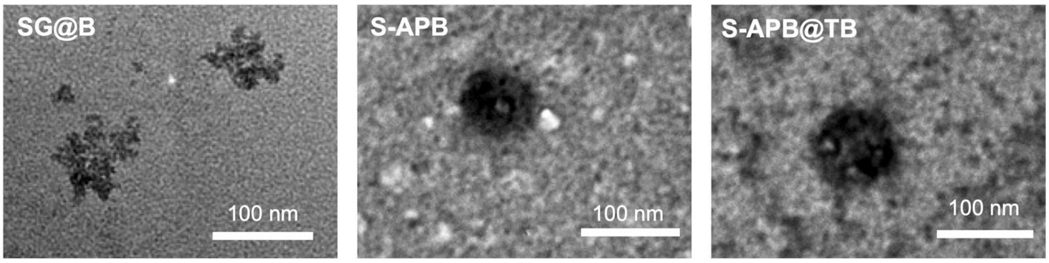

Figure S1. Transmission electron microscopy (TEM) analysis of SG@B, S-APB, and S-APB@TB. See text for details.

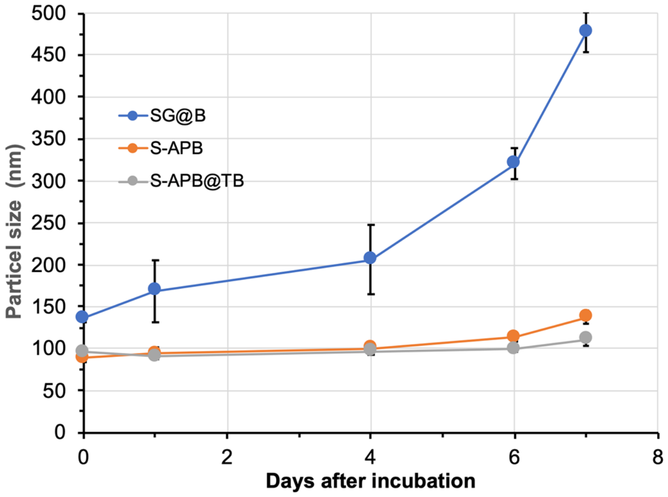

Figure S2. Changes in particles of SMA boron compounds after incubation at room temperature. The particle sizes were measured by dynamic light scattering (DLS). See the text for further details.

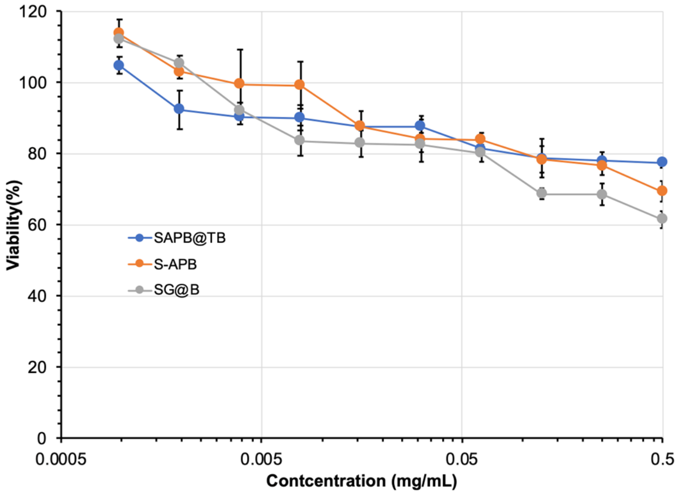

Figure S3. In vitro cytotoxicity of SMA boron compounds in Vero cells. See the text for further details.
